# Environmental Chemical Exposures and Mitochondrial Dysfunction: a Review of Recent Literature

**DOI:** 10.1007/s40572-022-00371-7

**Published:** 2022-07-28

**Authors:** Aalekhya Reddam, Sarah McLarnan, Allison Kupsco

**Affiliations:** 1 Department of Environmental Health Sciences, Mailman School of Public Health, Columbia University, 722 W 168th St, New York, NY 10032, USA

**Keywords:** Mitochondrial dysfunction, mtDNA, Environmental chemicals, Oxidative stress, Heteroplasmy

## Abstract

**Purpose of Review:**

Mitochondria play various roles that are important for cell function and survival; therefore, significant mitochondrial dysfunction may have chronic consequences that extend beyond the cell. Mitochondria are already susceptible to damage, which may be exacerbated by environmental exposures. Therefore, the aim of this review is to summarize the recent literature (2012–2022) looking at the effects of six ubiquitous classes of compounds on mitochondrial dysfunction in human populations.

**Recent Findings:**

The literature suggests that there are a number of biomarkers that are commonly used to identify mitochondrial dysfunction, each with certain advantages and limitations. Classes of environmental toxicants such as polycyclic aromatic hydrocarbons, air pollutants, heavy metals, endocrine-disrupting compounds, pesticides, and nanomaterials can damage the mitochondria in varied ways, with changes in mtDNA copy number and measures of oxidative damage the most commonly measured in human populations. Other significant biomarkers include changes in mitochondrial membrane potential, calcium levels, and ATP levels.

**Summary:**

This review identifies the biomarkers that are commonly used to characterize mitochondrial dysfunction but suggests that emerging mitochondrial biomarkers, such as cell-free mitochondria and blood cardiolipin levels, may provide greater insight into the impacts of exposures on mitochondrial function. This review identifies that the mtDNA copy number and measures of oxidative damage are commonly used to characterize mitochondrial dysfunction, but suggests using novel approaches in addition to well-characterized ones to create standardized protocols. We identified a dearth of studies on mitochondrial dysfunction in human populations exposed to metals, endocrine-disrupting chemicals, pesticides, and nanoparticles as a gap in knowledge that needs attention.

## Introduction

The mitochondrion is a fundamental component of the cell that plays a vital part in energy metabolism. In addition to generating energy, mitochondria are also important in multiple cell signaling cascades, metabolite generation, the homeostasis of various minerals and lipids, calcium storage, the immune response, the synthesis of steroids and heme groups, and apoptosis [1–5]. Given these diverse functions, mitochondria are a critical component of cellular homeostasis and survival.

Despite the various roles they perform within the cell, mitochondria are particularly vulnerable to damage. This is due in part to their proximity to reactive oxygen species (ROS). Oxidative phosphorylation, the main source of ATP generation, occurs in the inner mitochondrial membrane [6]. During this process, electrons leak from complexes I, II, and III and react with oxygen to form superoxide. The superoxide radical is then converted to hydrogen peroxide by superoxide dismutase, and together, hydrogen peroxide and superoxide are considered mitochondrial ROS [7, 8, 9]. Due to the proximity of its production, excess ROS can result in damage to mitochondrial biomolecules, induce mitochondrial DNA mutations, alter membrane permeability and structure, and change calcium ion (Ca^2+^) homeostasis [8, 10, 11]. Damage to mitochondrial DNA (mtDNA) is particularly concerning, as the mitochondria have reduced DNA repair capacity in comparison to the nucleus [12]. This is likely due to the reliance on polymerase γ for both replication and repair of mtDNA and a limited repair mechanism, primarily base excision repair, when dealing with mtDNA damage [13, 14]. This is significant because persistent mtDNA damage can have further downstream effects on the mitochondrion.

Due to their susceptibility to damage, mitochondria are highly sensitive to environmental toxicants. The charged difference between the mitochondrial matrix and the cytosol allows for positively charged and lipophilic chemicals to accumulate within the mitochondrial matrix [15, 16]. The damage caused by these chemicals within the mitochondria can manifest in multiple ways. Often, the damage leads to the disruption of the mitochondrial electron transport chain (ETC), which results in excess generation of ROS, and decreased ATP levels [7, 17]. Other types of damage can include dysregulation of Ca^2+^, changes in membrane permeability, and structural damage to the mitochondria [18, 19]. The different types of damage interact to exacerbate detrimental effects and can result in cell death. Hence, the goal of this review is to characterize the effect of various environmental toxicants on mitochondrial dysfunction, focusing on human population research published within the past 5 years when available. Tables 1 and 2 summarize the literature cited in this review in human populations and experimental studies, respectively.

## Mitochondrial Biomarkers for Environmental Health

**Fig. 1:**
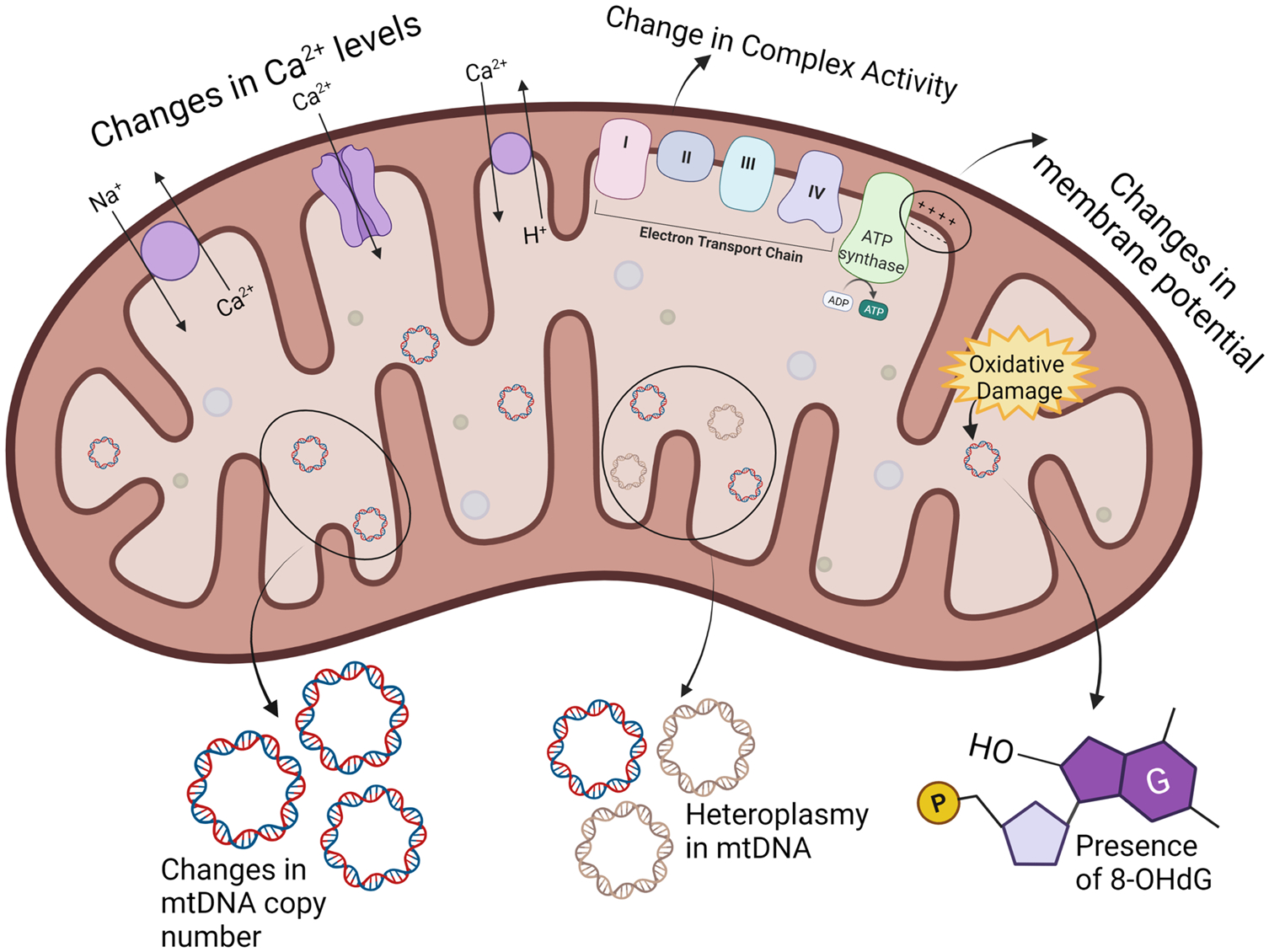
Common biomarkers used to identify and measure mitochondrial dysfunction. mtDNA mitochondrial DNA, Ca^2+^ calcium ions, 8-OHdG 8-oxo-2′-deoxyguanosine

Given the importance of the mitochondria and its susceptibility to damage, there is a growing need for sensitive biomarkers to detect mitochondrial dysfunction from environmental toxicants (Fig. 1). One of the most common biomarkers used in human population studies is changes in the mtDNA copy number (mtDNAcn). mtDNAcn is the number of mitochondrial genomes in a cell, and is positively correlated with the size and the number of mitochondria [20]. Each cell contains hundreds to thousands of mitochondria, each of which contains many copies of the mitochondrial genome. mtDNAcn can change depending on the energetic demands of the cells. For instance, muscle cells contain around 7000 copies of mtDNA per cell, which is higher compared to that of cells with a lower metabolic capacity [21]. Under environmental stressors, significant changes in mtDNAcn may indicate a biological response to excess ROS production and mtDNA damage and dysfunction [22, 23]. In fact, changes in mtDNAcn are associated with neurodegenerative, cardiovascular, and chronic kidney diseases, making them a relevant biomarker of mitochondrial dysfunction [24, 25, 26]. Moreover, measurement of mtDNAcn uses relatively simple techniques, making it an accessible biomarker for large human population studies [24, 27]. However, the mtDNAcn biomarker has some limitations. Conflicting associations have been observed in human population studies between chemical exposures and mtDNAcn which may be attributed to population characteristics, as well as the exposure concentration and duration. Furthermore, both an excess and a dearth of mtDNA can represent mitochondrial dysfunction, so consistency in the direction of effect across studies may not be informative. Additionally, significant variations between individuals and within an individual’s cell-specific mtDNAcn have been detected, which may be due to the various biological states that can lead to either an increase or a decrease in mtDNAcn [30•]. In particular, the magnitude and duration of oxidative stress and damage within the mitochondria may lead to varying responses in mtDNAcn. For instance, mitochondrial insult may initially result in mtDNA replication to compensate for the damage, leading to an increased copy number. However, it is also possible that past a certain threshold, the mitochondria are no longer able to compensate for the damage, leading to mitochondrial membrane permeability and apoptosis, which results in a decrease in the copy number [28, 29]. These different reasons give rise to the concern than the mtDNAcn values may be over interpreted [30•].

Heteroplasmy is another mitochondrial biomarker that describes the proportion of mutated mtDNA within a cell and may be used to indicate the severity of damage to the mitochondria [31, 32]. While a small amount of heteroplasmy (< 1%) in the mtDNA is normal, when the mtDNA undergoes damage, it may alter mitochondrial gene expression, leading to a higher proportion of mutations [32]. Hence, toxicant-induced mitochondrial damage may lead to a higher mtDNA mutation load, i.e., increased heteroplasmy, making it a relevant biomarker. In fact, recently published literature has demonstrated that heteroplasmy can be measured in human populations and is associated with changes in birth outcomes, respiratory functions, blood pressure, and depressive symptoms [33••, 34–36]. Heteroplasmy can also provide insight into mtDNA function through examination of heteroplasmic sites in coding regions [37]. However, for a biochemical defect to be detected, the proportion of mutated DNA must exceed a threshold level, and each cell, tissue, organ, and person has its own individual threshold, making it hard to compare across different populations [32, 38]. As a consequence, not many studies use heteroplasmy as a biomarker to measure the response to environmental toxicant exposure.

The mitochondrial respiratory chain is made up of five transmembrane enzyme complexes that work together with electron transfer carriers, ubiquinone, and cytochrome c, to produce ATP during oxidative phosphorylation. These complexes may be a target of environmental toxicants that alter their expression, concentration, or maximum activity [39]. During the process of oxidative phosphorylation, the complexes aid in the maintenance of an electrochemical gradient through a series of redox reactions. This electrochemical gradient generates the mitochondrial membrane potential and is an essential component of energy production. Either through the disruption of the complexes, perturbation of the electron transfer carriers or proteins, and/or damage to the membranes, external chemicals can alter the membrane potential, which may affect ATP and induce cell death [40, 41]. Changes in both the activity of the respiratory chain complexes and membrane potential are useful biomarkers because they help elucidate the mechanisms of toxicant-induced mitochondrial dysfunction. However, these measurements often require large quantities of fresh samples, which are beyond the capabilities of most cohort studies. Furthermore, a significant limitation is that the probes often used to measure these changes can be affected by the cellular membrane potential, mitochondrial pH, and changes in ATP production [41–44]. Nonetheless, more techniques are being developed to measure these mitochondrial bioenergetics functions in humans [45••].

Changes in oxidative phosphorylation, among other mitochondrial defects, often have downstream effects that are also commonly measured as biomarkers. The oxidation of guanine in mtDNA and the subsequent formation of 8-hydroxy-2′-deoxyguanosine (8-OHdG) is one of the main forms of free radical–induced DNA lesions [46]. High concentrations of mitochondrial 8-OHdG are indicative of oxidative DNA damage, and therefore are a common biomarker used to measure mitochondrial dysfunction [47]. Exposure to environmental toxicants can often lead to higher concentrations of ROS within the mitochondria and is associated with higher concentrations of 8-OHdG. The assays used to measure 8-OHdG are well established and are widely used to represent mitochondrial dysfunction in human populations. However, 8-OHdG is also detected in nuclear DNA, so mtDNA often needs to be separated prior to quantification. Additionally, there have been discrepancies between chromatographic and immunoassay approaches used to measure 8-OHdG within human samples [49].

Ca^2+^ levels play an important role in membrane potential regulation, ROS homeostasis, and oxidative phosphorylation within the mitochondria [50]. As a consequence, impaired mitochondrial Ca^2+^ transfer alters the production of ATP and downregulates mitochondrial metabolism, while high concentrations of mitochondrial Ca^2+^ suggest a disruption of the electrochemical gradient [50, 51]. Toxicant-induced overload of Ca^2+^ concentrations is associated with oxidative stress, a collapse in membrane potential, and eventually cell death [52]. While Ca^2+^ levels in in vitro models are commonly used to measure mitochondrial dysfunction, an important consideration is that this assay is unable to differentiate if toxicant-induced effects were a cause or consequence of the phenotype [39]. Additionally, there have been discrepancies in the Ca^2+^ levels measured using fluorescent dyes and genetically encoded calcium indicators, which may be attributed to the fact that mitochondria from different cell types uptake Ca^2+^ in different concentrations, making it hard to cover the full range using one type of sensor [48].

In addition to these measures of mitochondrial dysfunction, the alteration of cardiolipin is an emerging mitochondrial biomarker. Cardiolipin is a mitochondrion-exclusive phospholipid and plays an important role in mitochondrial protein transport, membrane morphology, cellular signaling, and bioenergetics [53, 54•]. While there has yet to be research examining associations between chemical exposure and cardiolipin levels, studies have found associations between cardiolipin alterations and diseases in human populations [54•, 55]. This suggests that it might be a relevant biomarker to account for when examining mitochondrial dysfunction.

Additionally, the presence of circulating cell-free mitochondria in blood may serve as an alternative matrix for the biomarkers discussed above. Cell-free mitochondria are the presence of whole and functioning mitochondria out of the cell, which has been detected within human blood [56•, 57]. In addition to whole mitochondria, cell-free mtDNA fragments are also detected in human blood, either encapsulated within extracellular vesicles or free-circulating. While the mechanisms and functions of cell-free mitochondria are relatively unknown, elevated levels of plasma cell-free mtDNA are associated with stress, inflammatory diseases, cancers, and sepsis in human populations [58•, 59, 60]. The emergence of standardized ways of measuring this biomarker may allow for wider use when looking at associations with toxicant-induced mitochondrial damage. The use of mitochondrial biomarkers in human population and experimental studies has provided great insight into the impact of environmental agents on mitochondrial function and health.

## Known Mitochondrial Disruptors

Much of our present knowledge on the critical role of mitochondria in health comes from the few chemicals whose mechanisms of toxicity on the mitochondria are well characterized. Acute poisoning from these highly specific mitochondrial toxicants leads to nausea, headaches, seizures, cardiac failure, and, in extreme cases, death. Cyanide is a potent mitochondrial inhibitor that binds to complex IV, specifically the a3 portion of cytochrome oxidase, within the ETC [61]. From there, cyanide competes with oxygen and binds to the Fe-Cu center which inhibits activity and energy production [62]. Rotenone, a pesticide and insecticide, is another mitochondrial inhibitor that affects the electron transfer from the Fe-S centers in complex I. This leads to the inhibition of oxidative phosphorylation and consequently a limited production of ATP, which further induces apoptosis in cells. Moreover, rotenone-induced apoptosis is closely related to mitochondrial ROS formation which may cause mitochondrial damage [63, 64]. Azidothymidine is an anti-HIV drug that accumulates within the mitochondrial intermembrane space where it disrupts the ATP/ADP translocator and enhances the production of ROS [65, 66]. Doxorubicin is an anticancer drug that also generates ROS; however, it does so by interacting with complex I and the proteins involved in oxidative phosphorylation [67, 68]. The resulting oxidative stress then goes on to cause mitochondrial injury and apoptosis. Lastly, exposure to benzene, a common industrial chemical and environmental toxicant, consistently increases mtDNAcn and alters mitochondrial pathways, possibly in response to the oxidative stress caused by benzene within the mitochondria [69–72]. Among all these classic mitochondrial disruptors, a common theme is disruption of energy production and oxidative stress. Understanding the well-established mechanisms of mitochondrial disruption caused by these chemicals has allowed researchers to investigate the role of other ubiquitous and well-known toxicants on mitochondrial dysfunction.

## Polycyclic Aromatic Hydrocarbons

Polycyclic aromatic hydrocarbons (PAHs) are a class of compounds that are common byproducts of incomplete combustion. They are frequently detected following incineration of industrial, domestic, and agricultural products and emissions from vehicles [73]. Once emitted, PAHs may bind to or form small particles in the air which subsequently lead to human exposure. PAHs are highly lipophilic toxicants and therefore readily accumulate in the mitochondria due to their high lipid content [74]. In fact, PAHs are also shown to preferentially bind to the mtDNA at 40–90 times greater than nuclear DNA [74, 75]. Moreover, the mitochondrial cytochrome P450 system may bioactivate PAHs to make them more toxic in the organelle [76]. PAHs may also be activated through mitochondrial aldo–keto reductase and/or manganese superoxide dismutase which causes the production of ROS [77]. In vitro studies have shown that exposure to PAHs triggers mitochondrial oxidative damage in blood lymphocytes and affects the mitochondrial redox machinery which leads to higher concentrations of ROS [78]. This excess generation of ROS and associated oxidative stress within the mitochondria may act as a regulator of the mtDNAcn [29, 79], leading to mtDNAcn changes in populations exposed to PAHs.

The literature examining the associations between PAH exposure and mtDNAcn within human populations is inconclusive. Higher urinary PAH metabolites were associated with higher mtDNAcn in peripheral blood samples of asphalt workers [80] and in leukocytes of coke oven workers [77]. Urinary PAH metabolites were also positively associated with increased peripheral blood mtDNAcn in an urban population in China [81]. Prenatal exposure to PAHs measured through maternal urinary metabolites was associated with increased mtDNAcn in cord blood in China [82]. Conversely, other studies have also shown negative associations between PAH exposure and mtDNAcn. Increased urinary PAH metabolites were associated with decreased mtDNAcn in college student sperm samples [83] and leukocytes of non-smoking women [84]. Occupational exposures to PAHs in different coke oven workers showed significantly lower mtDNAcn in peripheral blood compared to the control groups [85•, 86, 87]. This relationship was also detected in the blood of individuals that lived in homes with a higher PAH concentration in their house dust [88]. The differences in mtDNAcn may be attributed to varied exposure levels between the different studies; however, because exposures to PAHs were measured in different matrices, we cannot directly compare across studies.

## Particulate Air Pollutants and Black Carbon

Air pollution is a complex mixture that consists of a variety of physical and chemical components depending on the sources [89]. While airborne PAHs are due to combustion of fuel sources, the presence of other chemical substances, gases, or particulate matter within the air is attributed primarily to vehicle exhaust and industry emissions. In this section, we will focus on the compounds, other than PAHs, that have clearly displayed toxic effects on the mitochondria. Mitochondria are susceptible to air pollutants particularly due to their lack of repair capacity and their enhanced vulnerability to ROS. Experimental studies have shown that exposure to air pollutants leads to oxidative stress, changes in mitochondrial membrane potential, and decreases in mtDNAcn in cells [90–92] and lower mtDNAcn, lower mitochondrial consumption rate, and mitochondrial structural abnormalities in mice [92, 93].

Air pollutants are some of the most well-studied exposures in relation to mitochondria in humans. Studies have shown that increased prenatal exposure to particulate matter (PM) was associated with increased levels of mitochondrial urinary 8-OHdG in maternal and umbilical cord blood, suggesting oxidative stress within the mitochondria [94]. Moreover, during the air quality intervention for the Beijing Olympic Games, a reduction in ambient air pollutant levels led to a significant decreased in urinary 8-OHdG levels in schoolchildren [95].

Similar to PAHs, particulate air pollutants have a varied effect on mtDNAcn, possibly as a response to the excess ROS within the mitochondria. Increased PM_2.5_ (PM with a diameter of 2.5 μm or less), PM_10_ (PM with a diameter of 10 μm or less), and black carbon (BC) exposure was associated with a decrease in mtDNAcn in the blood of an elderly Flemish truck driver population and leukocytes of an elderly Belgian population [96–98]. Moreover, studies have also shown that prenatal exposure to NO_2_, PM_10_, and PM_2.5_ are associated with decreased placental mtDNAcn [84, 98, 99, 100] and cord blood mtDNAcn [101, 102]. Other studies, however, have shown that occupational PM exposure was associated with increased whole-blood mtDNAcn in steel workers [103, 104] and BC exposure was positively associated with whole-blood mtDNAcn in older adults [105]. Exposure levels, duration of exposure, and life stages of the participants in these studies are highly varied, which may contribute to differences in study findings. Lastly, in addition to changes in mtDNAcn, PM_2.5_ and NO_2_ have shown to be positively associated with mtDNA methylation in blood and placenta [104, 106•, 107] and DNA methylation in mitochondrion-related genes in umbilical cord blood [108]. Moreover, PM_2.5_ was associated with an increase in heteroplasmy on genes coding for NADH dehydrogenase and subunits for ATP synthase in mtDNA [109]. PM_10_ exposure was also associated with transcriptomic pathways related to mitochondrial genome maintenance, ETC, and tricarboxylic acid (TCA) cycle in whole blood, suggesting that the pathways were upregulated to compensate for the PM-induced damage [110]. Prenatal exposure to PM_2.5_ has also been shown to be positively associated with a decrease in mitochondrial function in blood and placenta [106•, 107].

## Heavy Metals

Heavy metals, specifically cationic metals, are shown to preferentially accumulate within the mitochondria through the calcium transporter due to their similarity to the Ca^2+^ ion [111]. Moreover, the mitochondrial membrane contains unsaturated lipids which enhance its susceptibility to metals, such as arsenic (As), compared to other organelles [112]. Human population studies have shown that exposure to manganese (Mn), aluminum (Al), and lead (Pb) in the prenatal period has resulted in an increase in mtDNAcn in cord blood, and exposure to Pb was associated with an increase in maternal mtDNAcn [113•, 114, 115•, 116]. Conversely, exposure to thallium and As was associated with a decrease in mtDNAcn in cord blood leukocytes, and magnesium (Mg) exposure was associated with decreased maternal and cord blood mtDNAcn [116–118]. Smith et al. (2021) also reported a non-linear relationship between prenatal Mg exposure and cord blood mtDNAcn, as well as between barium, Pb, and mercury (Hg) exposure and maternal mtDNAcn. Interestingly, they did not find any significant associations between As, cadmium (Cd), cesium, Mn, selenium, and zinc exposure and mtDNAcn [116].

Much of the literature examining the effect of metals on mitochondrial dysfunction details experiments conducted in in vitro and animal models, and therefore, this section of the review, as well as for the following chemical classes, will focus on elucidating mechanisms behind this toxicity that might be relevant to humans. The most common dysfunction induced by heavy metals is the production of elevated mitochondrial ROS. The Fenton reaction, where transition metals such as iron and copper (Cu) catalyze the generation of hydroxyl radicals from hydrogen peroxide, has been commonly implicated in the production of ROS [119, 120]. Cu, Cd, Pb, Mn, Hg, As, and Al have all shown to increase ROS which in turn triggers mitochondrial dysfunction and subsequent apoptotic and autophagic death in both in vitro systems and rodent models [62, 111, 121–129]. In human populations, high Cd exposure was associated with higher 8-OHdG and citrate (a urinary metabolite associated with mitochondrial metabolism) levels [130].

In addition to producing excess ROS, Cu, Cd, and As decreased the transmembrane potential and ATP levels in human cell lines and rats [111, 122, 124, 128, 131, 132]. This is possibly through the inhibition of ADP, which induces ion permeability of the inner mitochondrial membrane [133]. Once the membrane potential is lost, cytochrome c is released and caspases may be activated, leading to apoptosis of the mitochondria [128, 134]. In addition, Cd treatment also inhibits mitochondrial respiratory chain enzymes within human osteoblasts [122] and leads to organelle swelling causing the inhibition of respiration in rats [135].

Another mechanism of toxicity for other heavy metals such as Pb, Mn, As, and Hg is via Ca^2+^-dependent signaling pathways. Mitochondria have been implicated as major sites for Pb^2+^ and Mn^2+^ accumulation [127, 136], following which both Pb^2+^ and Mn^2+^ can substitute for Ca^2+^ in the Ca^2+^ uniporter and TCA cycle dehydrogenases, respectively, and cause Ca^2+^ dysregulation in the mitochondria [62]. This in turn induces Ca^2+^ efflux, which leads to decreased NADH levels in the mitochondria and eventually apoptosis.

## Endocrine-Disrupting Chemicals

Endocrine-disrupting chemicals (EDCs) are a class of compounds that modulate hormone action primarily by mimicking naturally occurring hormones, binding to their respective receptors and changing downstream pathways [137]. There are a wide variety of chemicals that are classified as EDCs, including phthalates, parabens, and bisphenols. These are commonly used as plasticizers in consumer products but are also used in pharmaceuticals, cosmetics, and personal care products [138]. As EDCs affect different cellular processes, including those related to energy production and utilization, it is thought that EDC disruption of energy homeostasis may be associated with mitochondrial dysfunction [139•].

Exposures to phthalates and bisphenols have been shown to be associated with changes in mtDNA methylation [140]. Specifically, EDCs such as alkylphenol 4-nonylphenol (NP), di(2-ethylhexyl) phthalate (DEHP), monoethylhexyl phthalate (MEHP), and bisphenol A (BPA) are associated with elevated oxidative stress through increased ROS production, changes in redox homeostasis, and production of extracellular superoxide [139•, 140–146]. This in turn affects the mtDNAcn as described for toxicants above. Human studies have shown that exposure to phthalates is positively associated with mtDNAcn in sperm and bisphenol S (BPS) is positively associated with mtDNAcn in children [147•, 148].

In addition to oxidative stress, studies have shown that BPA exposure was associated with a decrease in mitochondrial respiratory complex activity and consequently a decrease in mitochondrial membrane potential and ATP production in human lymphoblasts and rat models [146, 149, 150]. BPA and BPS may also alter the expression of regulatory genes related to mitochondrial energy metabolism, mitochondrial fusion and division, and mitochondrial fatty acid metabolism in rats [145, 149, 151]. Additionally, DEHP exposure is associated with mitochondrial ultrastructural abnormalities in quail [141].

## Pesticides

Pesticides are a large class of chemical compounds with a wide range of properties that lend themselves to different modes of action when inducing mitochondrial toxicity. Organophosphate (OP) and organochlorine (OC) pesticides are classes of chemicals that are highly lipophilic and can therefore easily enter and accumulate within the mitochondria similar to PAHs. In fact, OP pesticides with hydrophobic properties have an increased mitochondrial translocator protein–binding affinity [152]. Once in the mitochondria, both OP and OC pesticides have been shown to reduce the mitochondrial membrane potential, produce mtDNA damage, promote oxidative damage, and reduce mitochondrial ATP in cell lines and zebra fish [152, 153, 156]. In addition to these other mechanisms, Budnik et al. (2013] also showed that exposure to OC pesticides was significantly associated with elevated serum levels of circulating mtDNA, suggesting decreased integrity of mtDNA in exposed individuals. Additionally, prenatal exposure to benzothiazoles, a class of compounds that are used as fumigants, is associated with changes in mtDNAcn in cord blood [158]. In this study, investigators observed a positive association with exposure measured in the first trimester, which was then reversed in the third trimester.

Paraquat and atrazine, two widely used pesticides, induce mitochondrial toxicity through very similar mechanisms. Both paraquat and atrazine produce ROS which induces mitochondrial toxicity [159, 160]. Both compounds adversely affect the electron transfer within the ETC to form a superoxide anion which forms an excess of ROS in various animal systems [159–163]. Exposure to paraquat and atrazine has also been shown to decrease mitochondrial membrane potential in pigs and mice [160, 164]. In addition to these mechanisms, atrazine has been shown to activate the mitochondrial unfolded protein response, as well as increase mitochondrial damage and vacuolar degeneration, and decrease mitochondrial cristae and volume density in *Caenorhabditis elegans* [163].

## Nanomaterials

Nanomaterials are particles that range from 1 to 100 nm that may be formed naturally or engineered. Nanomaterials are found in numerous consumer products including cosmetics, tires, and electronics. Once in the body, due to their small size, nanomaterials are easily transported across cell membranes where they can accumulate within the mitochondria [165, 166•] and lead to the disruption of the mitochondrial membrane potential and structure [166•, 167]. Nanomaterials are distinct from the previous classes of chemicals in that they are primarily physical rather than chemical stressors. Studies have shown that exposure to silver nanoparticles, hydroxyapatite nanoparticles, cadmium telluride quantum dots, graphene, fullerene, and carbon nanotubules leads to a significant decrease in mitochondrial membrane potential and ADP-induced depolarization through increased permeability of the mitochondrial inner membrane and induction of mitochondrial permeability transition [168–172] in both human and rat in vitro systems. Exposure to nanomaterials also leads to increased intracellular Ca^2+^ levels and over-production of ROS in human cells [171, 172, 173]. They are also associated with a change in levels and activities of enzymes of the ETC [171, 174]. In addition to the changes within the ETC, the presence of iron-rich nanoparticles and graphene oxide in mitochondria is associated with deformed cristae and ruptured membranes in human heart samples and zebra fish models [175, 176]. This in vitro evidence suggests that nanoparticles are associated with mitochondrial toxicity, and therefore could be important for human health effects. Hence, more research in human populations is key towards understanding the mitochondrial health impacts of nanoparticles.

## Conclusion

**Fig. 2:**
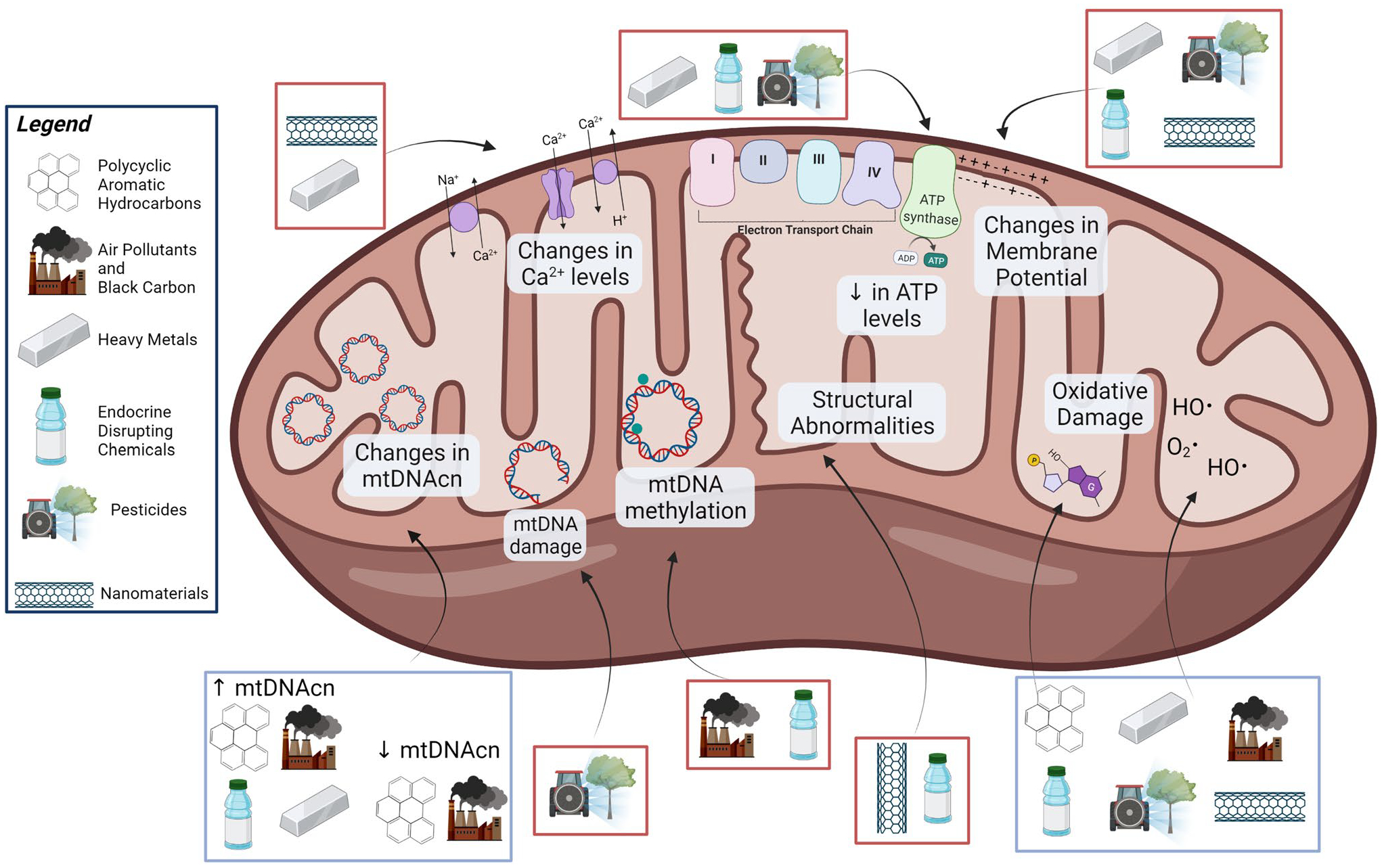
The environmental toxicant–induced mitochondrial dysfunction pathways discussed within this review. Blue boxes outlining the environmental toxicants represent associations shown in both human populations and experimental models, whereas red boxes represent associations found only in experimental models

A large body of human population and experimental research suggests that multiple classes of environmental toxicants can induce mitochondrial stress and disrupt mitochondrial function (Fig. 2, Tables 1 and 2). Several chronic diseases are characterized by system- or organ-specific mitochondrial dysfunction. As discussed throughout, disparate toxicants can induce common types of mitochondrial damage and responses. For instance, excess production of ROS, a ubiquitous response across different chemical classes, is commonly tied to other mitochondrial biomarkers and dysfunction such as alterations of mitochondrial membrane permeability, calcium homeostasis, and ATP production [177–179]. Moreover, the presence of excess ROS within the mitochondria can induce a positive feedback loop in the mitochondrial environment, leading to more ROS release [180, 181]. Superfluous ROS may affect the normal functioning of mitochondria, cells, and organisms and is tied to cardiovascular diseases [182], autism spectrum disorder [183], neurodegenerative diseases [181, 184], obesity [185], and diabetes [178]. Another common response to the different forms of mitochondrial damage is a decrease in mitochondrial energetics, as demonstrated through reduction in ATP levels and oxygen consumption. This decrease has also been associated with the onset of chronic kidney diseases [186], heart diseases [187, 188], neurodegenerative diseases [189–191], liver diseases [192], and diabetes [193]. Lastly, persistent mtDNA damage caused by chemical exposure may inhibit replication, RNA transcription, and mitochondrial function. Therefore, it is associated with neurodegenerative diseases [194, 195], cardiovascular diseases [196, 197], liver diseases [198], inflammatory diseases [199], kidney diseases [200, 201], and obesity [202].

A wealth of experimental evidence indicates the ability of environmental toxicant exposures, such as PAHs and air pollutants, to induce mitochondrial dysfunction. However, there is a greater need for more studies examining the role of additional chemicals such as heavy metals, EDCs, pesticides, and nanomaterials in mitochondrial dysfunction within human populations. Understanding the associations between toxicant exposure and mitochondrial dysfunction in humans may help elucidate potential mechanisms through which these chemicals induce toxicity. Moreover, recognizing these mechanisms may aid in the development of therapeutics that target the mitochondrial dysfunction and prevent disease advancement [203, 204].

As described within this review, most of the human population studies linking exposure to mitochondrial dysfunction used blood or placental mtDNAcn as a biomarker. While changes in mtDNAcn can suggest mitochondrial dysfunction and may be associated with health outcomes [25, 96], they are not a perfect representation of mitochondrial content or biogenesis and there is inherent variability in copy number associated with the cell type composition within a tissue or biospecimen [30•]. Furthermore, the inconsistent directionality of changes in mtDNAcn may make it difficult to interpret the nature of the adverse effects. Additional research is needed to untangle the complex impacts of toxicants on mtDNAcn and their significance within human populations. Therefore, with the advent of new techniques and biomarkers such as cell-free mitochondria [56•, 205] and cardiolipin levels in blood [206], there is a need to apply these novel approaches and generate a standardized protocol to continue to characterize the mechanisms behind and consequences of toxicant-induced mitochondrial dysfunction.

## Figures and Tables

**Table 1:** Environmental toxicants and mitochondrial dysfunction measured in human population studies outlined in this review. Biospecimen column refers to the tissue the mitochondrial biomarker was measured in

Compound	Population	Location	Study design	Biospecimen	Results	Citation
Polycyclic aromatic hydrocarbons						
Benzene	Workers	China	Cross-sectional	Plasma	↑ Oxidative stress	Rothman et al. 2021 [69]
	Workers	Italy	Cross-sectional	Whole blood	↑ Copy number	Carugno et al. 2012 [70]
	Workers	China	Cross-sectional	Whole blood	↑ Copy number	Shen et al. 2008 [72]
Benzo[a]pyrene	Female adults	China	Cross-sectional	Leukocytes	↓ Copy number	Wong et al. 2017 [84]
PAH mixture	Workers	China	Cross-sectional	Peripheral blood	↓ Copy number	Du et al. 2020 [85]
	Workers	China	Cross-sectional	Peripheral blood	↓ Copy number	Zhao et al. 2020 [86•]
	Workers	China	Cross-sectional	Leukocytes	↓ Copy number	Duan et al. 2020 [87]
	Male workers	Sweden	Cross-sectional	Peripheral blood	↑ Copy number	Xu et al. 2018 [80]
	Male adults	China	Cross-sectional	Sperm	↓ Copy number	Ling et al. 2017 [83]
	Workers	Poland	Cross-sectional	Blood lymphocytes	↑ Copy number	Pavanello et al. 2013 [77]
	Adults	Belgium	Cross-sectional	Blood	↓ Copy number (winter only)	Pieters et al. 2013 [88]
PAH metabolite mixture	Pregnancy (mother/newborn)	China	Longitudinal	Cord blood	↑ Copy number	Cao et al. 2020 [82]
	Urban adults	China	Cross-sectional	Whole blood	Direction in copy number change dependent on time since exposure	Hou et al. 2019 [81]
Particulate air pollution						
PM	Male workers	Italy	Cross-sectional	Whole blood	↑ Copy number	Hou et al. 2010 [103]
PM_2.5_	Pregnancy (mother/newborn)	USA	Longitudinal	Placenta	↑ mtDNA non-synonymous mutation load	Brunst et al. 2022 [109]
	Pregnancy (mother/newborn)	USA	Longitudinal	Peripheral blood mononuclear cells	Altered mitochondrial respiration	Frye et al. 2021 [107•]
	Pregnancy (mother/child up to age 8)	Europe and North America	Longitudinal	Cord blood	Association with methylation of nuclear encoded mitochondrial genes	Gruzieva et al. 2017 [108]
	Pregnancy (mother/newborn)	Mexico	Longitudinal	Cord blood	↓ Copy number	Rosa et al. 2017 [101]
	Elderly males	USA	Retrospective	Blood lymphocytes	↓ Copy number	Peng et al. 2017 [100]
	Elderly	Belgium	Cross-sectional	Leukocytes	↓ Copy number	Pieters et al. 2016 [97]
	Pregnancy (mother/newborn)	Belgium	Longitudinal	Placenta	↑ mtDNA methylation and ↓ copy number	Janssen et al. 2015 [106]
PM_10_	Adults	Belgium	Cross-sectional	Whole blood	Sex-dependent altered gene expression of mitochondrial genes	Winckelmans et al. 2017 [110]
PM_2.5_ and PM_10_	Pregnancy (mother/newborn)	Belgium	Longitudinal	Maternal and cord blood	↑ mitochondrial 8-OHdG	Grevendonk et al. 2016 [94]
Metal-rich PM_1_	Male workers	Italy and China	Cross-sectional	Peripheral blood	↑ mtDNA methylation	Byun et al. 2013 [104]
NO_2_ and black carbon	Elderly	Belgium	Repeated-measure	Whole blood	↓ Copy number	Bai et al. 2018 [98]
NO_2_	Pregnancy (mother/newborn)	Belgium and Spain	Prospective	Placenta	↓ Copy number	Clemente et al., 2016 [99]
Black carbon	Elderly males	USA	Repeated-measure	Whole blood	↑ Copy number	Zhong et al. 2016 [105]
	Workers	China	Repeated-measure	Whole blood	↓ Copy number	Hou et al. 2013 [96]
PM_2.5_ and black carbon	Children	China	Repeated-measure	Urine	↑ MDA and 8-OHdG	Lin et al. 2015 [95]
Metals						
Magnesium	Pregnancy (mother/newborn)	USA	Prospective	Maternal and cord blood	↓ Copy number, non-linear relationship with cord blood copy number	Smith et al. (2021) [116•]
Arsenic	Pregnancy (mother/newborn)	China	Prospective	Cord blood	↓ Copy number	Song et al. 2020 [118]
Manganese	Pregnancy (mother/newborn)	Mexico City	Prospective	Cord blood	Direction in copy number change dependent on maternal hemoglobin level	Kupsco et al. 2019 [114•]
Lead	Pregnancy (mother/newborn)	USA	Prospective	Maternal blood	↑ Copy number, non-linear relationship with copy number	Smith et al. 2021 [116•]
	Pregnancy (mother/newborn)	Mexico City	Prospective	Cord blood	↑ Copy number	Sanchez-Guerra et al. 2019 [113]
Aluminum	Pregnancy (mother/newborn)	China	Prospective	Cord blood	↑ Copy number	Liu et al. 2019 [115]
Thallium	Pregnancy (mother/newborn)	China	Prospective	Cord blood	↓ Copy number	Wu et al. 2019 [117]
Cadmium	Adults	England	Cross-sectional	Urine	↑ 8-OHdG	Ellis et al. 2012 [130]
EDCs						
Monocarboxy-isononyl phthalate	Male adults	USA	Cross-sectional	Sperm	↑ Copy number	Huffman et al. 2018 [147]
Pesticides						
Benzothiazoles	Pregnancy (mother/newborn)	China	Prospective	Cord blood	Direction in copy number change dependent on trimester	Chen et al. 2020 [158]
Halo alkane–based pesticides	Adults	Germany	Cross-sectional	Blood	↑ Circulating cell-free mtDNA and ↓ mtDNA integrity	Budnik et al. 2013 [157]
Nanoparticles						
Iron-rich nanoparticles	Children/young adults	Mexico City	Retrospective	Postmortem heart	↑ ROS and mitochondrial structural abnormalities	Maher et al. 2020 [175]

*mtDNA* mitochondrial DNA, *8-OHdG* 8-hydroxy-2′-deoxyguanosine, *ROS* reactive oxygen species, *MDA* malondialdehyde

**Table 2:** Environmental toxicants and their respective mitochondrial dysfunction measured in animal and in vitro studies outlined in this review. Biospecimen column refers to the tissue the mitochondrial biomarker was measured in

Compound	Species	Biospecimen	Dose/duration	Result	Citation
PAHS					
Benzo[a]pyrene	Human	Blood lymphocytes	10 μM for 1, 3, 6, 12, 24, 48, or 72 h	Altered expression of mitochondrial targeting microRNAs and epigenetic modifiers, and hypomethylation of mtDNA	Bhargava et al. 2020 [78]
	Human	Tk6 cells	0.05, 0.5, 5.0, 50, 500 μM for 24 h	↓ Copy number	Pieters et al. 2013 [88]
Heavy metals					
Cadmium	Human	Osteoblasts	65 μM for 24 or 48 h	↑ Oxidative stress, ↓ antioxidant gene expression, and ↓ MMP	Monteiro et al. 2018 [122]
	Human	PC12 cells	10, 50, 100, 500 μM for 3 or 24 h	Uncoupled cellular respiration	Belyaeva et al. 2012 [111]
	Guinea pig	Isolated heart, brain, liver mitochondria	0, 10, 20, 30, 40, 50 μM for 10 min	↑ ROS production and ↓ activity of complexes II and III	Wang et al. 2004 [121]
	Rat	Isolated liver mitochondria	1–100 μM for 1 min/stage	↑ Mitochondrial swelling	Belyaeva et al. 2002 [133]
	Rat	Isolated liver mitochondria	0–30 μM for 30 min	↑ Mitochondrial swelling, ↓ respiration, ↓ MMP, and ↓ preaccumulated Ca^2+^	Al-Nasser 2000 [135]
Aluminum	Human	PC12 cells	125–2000 μM for 48 h	↑ ROS and apoptosis, ↓ MMP, and catalase activity	Iranpak et al. 2019 [129]
Mercury	Human	PC12 cells	10, 50, 100, 500 μM for 3 or 24 h	Uncoupled cellular respiration	Belyaeva et al. 2012 [111]
Copper	Human	GC-1 cell line	0, 10, 50, 100 μM for 24 h	↓ MMP, ATP levels, and mitochondrial fission	Kang et al. 2019 [124]
	Rat	Isolated brain, liver, kidneys, heart mitochondria	500 and 1340 μM for 48 h	↓ MMP and ATP production, and altered mitochondrial structure	Borchard et al. 2018 [131]
	Rat	Isolated hippocampus mitochondria	50, 100, 200 mg/kg/day for 21 days	↑ ROS, ↑ mitochondrial swelling, ↑ lipid peroxidation, ↑ glutathione oxidation, ↑ outer membrane damage, ↓ MMP, ↓ cytochrome c oxidase activity, and ↑ ADP/ATP ratios	Behzadfar et al. 2017 [132]
	Human	PC12 cells	10, 50, 100, 500 μM for 3 or 24 h	Uncoupled cellular respiration	Belyaeva et al. 2012 [111]
Lead	Rat	Brain	220 ppm for 25 days	↑ Catalase activity and ↓ ALDH2 expression	Mattalloni et al. 2019 [126]
	Rat	Isolated brain mitochondria	0.2% in H_2_O for 37 days	↓ Enzyme activity and ↑ MDA levels	Gottipolu and Davuljigari 2014 [125]
	Yeast		0, 100, 250, 500, 1000 μM for 3 h	↑ ROS and ↑ mtDNA mutations	Sousa and Soares, 2014 [136]
Arsenic	Rat	Hippocampus	20 mg/kg for 21 days	↑ ROS, ↓ MMP, mitochondrial swelling, and release of cytochrome c	Keshavarz-Bahaghighat et al. 2018 [128]
Endocrine-disrupting compounds					
Di(2-ethylhexyl) phthalate	Quail	Liver	0, 250, 500, 1000 mg/kg/day for 45 days	↑ MDA, ↑ GSH and GST levels, ↓ antioxidant function, and ↑ mitochondrial structural abnormalities	Zhang et al. 2019 [141]
Bisphenol A	*C. elegans*		500 μM for 24 h	↑ Oxidative stress and mitochondrial dysfunction	Hornos Carneiro et al. 2020 [142]
	Rat	Liver	50 or 500 μg/kg/day for 20 wks	Dysregulated expression of ETC genes and altered expression of antioxidant genes	Azevedo et al. 2020 [151]
	Rat	Isolated liver mitochondria	40 μg/kg/day for ~ 42 days	↓ Complex I and III activity, ↓ ATP production, ↑ ROS, and cytochrome c release	Jiang et al. 2014 [149]
	Human	Lymphoblasts	0, 25, 50, 100 μM for ~ 12 h	↑ ROS, ↓ MMP, and ↑ copy number	Kaur et al. 2014 [146]
Nonylphenol	Rat	Pancreas	0, 20, 60, 180 mg/kg for 90 days	↑ ROS, ↓ MMP, and ↑ intracellular Ca^2+^	Li et al. 2017 [144]
Mono-2-ethylhexyl phthalate	Mouse	Leydig cells	1, 3, 10, 30, 90 μM for 48 h	↓ ATP production and ↑ ROS	Savchuk et al. 2015 [143]
Pesticides					
Mixed organochlorine pesticides	L6 myotube and zebra fish		Myotube: 0.5, 50, 5000 nmol for 48 h; zebra fish 0.15 and 75 nmol/L for 48 h	↑ ROS and ↓ mitochondrial quantity	Park et al. 2021 [155]
Dichloro diphenyl dichloroethylene	Mouse	Hepatocytes	DDE 1 mg/kg/day or HCH 10 mg/kg/day for 8 days	Changes in TCA metabolites, ↓ MMP, ↓ ATP levels, and ↓ oxygen consumption rate	Liu et al. 2017 [156]
Atrazine	*C. elegans*		0, 0.001, 0.01, 0.1, 1, 10 ng/L ~ 4.5 days	↑ ROS and activated mitochondrial unfolded protein response	Zhou et al. 2021 [164]
	Pig	Oocyte	0, 50, 100, 200, 500 μM for ~ 43 h	↑ ROS, ↓ MMP, and ↓ GSH production	Yuan et al. 2017 [160]
Paraquat	Human	Brain microvascular endothelial cells	1, 10, 100 μM for 24 h	↓ Complex I proteins	Tatjana et al. 2021 [162]
	Mouse	Cardiomyocytes	45 mg/kg for 48 h	↓ MMP	Wang et al. 2014 [164]
	Rat	Isolated brain mitochondria	30, 100, 300 μM for 10 min	↑ ROS	Drechsel et al. 2009 [161]
Nanoparticles					
Silver nanoparticles	Rat	Isolated liver mitochondria	40 or 80 nM for 10 min	↓ MMP, ↓ in ADP-induced depolarization, and ↓ respiratory control ratio	Teodoro et al. 2011 [168]
Cadmium telluride quantum dots	Human	Bronchial epithelial cells	20 μg/mL for 24 h	↑ Oxidative stress	Xu et al. 2019 [170]
	Human	Hepatocellular carcinoma HepG2 cells	10 mg/mL for 1 h	Enlarged mitochondria, disrupted ΔΨm, ↑ intracellular Ca^2+^, ↓ ATP, and ↑ mitochondrial biogenesis	
Pristine graphene	Human	U87 and HS-5 cells	0, 20, 50, 100, 200 μg/mL for 24 h	↑ ROS and ↓ MMP	Jaworski et al. 2019 [172]
Titanium dioxide nanoparticles	Human	HeLa cells	270 or 500 μg/mL for 1 h	↑ ROS	Jayaram et al. 2017 [173]
Hydroxyapatite	Rat	Hepatocytes	200–800 μg/mL for 24 h	↓ MMP, ↑ ROS, ↑ MDA, ↓ GSH, and ↓ complex I, II, and III activity	Xue et al. 2017 [174]
	Rat	Liver	50 mg/kg for 48 h	Mitochondrial swelling and ↓ succinate	
Graphene oxide	Zebra fish	Brain	0.01 and 0.1 μg/L for 24 h	↑ Oxidative stress and mitochondrial structural abnormalities	Ren et al., 2016 [176]

*mtDNA* mitochondrial DNA, *MMP* mitochondrial membrane potential, *ROS* reactive oxygen species, *ATP* adenosine triphosphate, *ADP* adenosine diphosphate, *ALDH2* aldehyde dehydrogenase, *MDA* malondialdehyde, *GSH* glutathione, *GS*T glutathione s-transferase, ETC electron transport chain, *TCA* tricarboxylic acid cycle
